# Assessment of productivity of hospitals in Botswana: a DEA application

**DOI:** 10.1186/1755-7682-3-27

**Published:** 2010-11-05

**Authors:** Naomi Tlotlego, Justice Nonvignon, Luis G Sambo, Eyob Z Asbu, Joses M Kirigia

**Affiliations:** 1 Department of Economics, University of Botswana, Private Bag UB 0022, Gaborone, Botswana; 2 School of Public Health, University of Ghana, P.O. Box LG 13, Legon, Ghana; 3 World Health Organization Regional Office for Africa, B.P. 06, Brazzaville, Congo

## Abstract

**Background:**

Botswana national health policy states that the Ministry of Health shall from time to time review and revise its organization and management structures to respond to new developments and challenges in order to achieve and sustain a high level of efficiency in the provision of health care. Even though the government clearly views assuring efficiency in the health sector as one of its leadership and governance responsibilities, to date no study has been undertaken to measure the technical efficiency of hospitals which consume the majority of health sector resources. The specific objectives of this study were to quantify the technical and scale efficiency of hospitals in Botswana, and to evaluate changes in productivity over a three year period in order to analyze changes in efficiency and technology use.

**Methods:**

DEAP software was used to analyze technical efficiency along with the DEA-based Malmquist productivity index which was applied to a sample of 21 non-teaching hospitals in the Republic of Botswana over a period of three years (2006 to 2008).

**Results:**

The analysis revealed that 16 (76.2 percent), 16 (76.2 percent) and 13 (61.9 percent) of the 21 hospitals were run inefficiently in 2006, 2007 and 2008, with average variable returns to scale (VRS) technical efficiency scores of 70.4 percent, 74.2 percent and 76.3 percent respectively. On average, Malmquist Total Factor Productivity (MTFP) decreased by 1.5 percent. Whilst hospital efficiency increased by 3.1 percent, technical change (innovation) regressed by 4.5 percent. Efficiency change was thus attributed to an improvement in pure efficiency of 4.2 percent and a decline in scale efficiency of 1 percent. The MTFP change was the highest in 2008 (MTFP = 1.008) and the lowest in 2007 (MTFP = 0.963).

**Conclusions:**

The results indicate significant inefficiencies within the sample for the years under study. In 2008, taken together, the inefficient hospitals would have needed to increase the number of outpatient visits by 117627 (18 percent) and inpatient days by 49415 (13 percent) in order to reach full efficiency. Alternatively, inefficiencies could have been reduced by transferring 264 clinical staff and 39 beds to health clinics, health posts and mobile posts. The transfer of excess clinical staff to those facilities which are closest to the communities may also contribute to accelerating progress towards the Millennium Development Goals related to child and maternal health.

Nine (57.1 percent) of the 21 hospitals experienced MTFP deterioration during the three years. We found the sources of inefficiencies to be either adverse change in pure efficiency, scale efficiency and/or technical efficiency.

In line with the report *Health financing: A strategy for the African Region*, which was adopted by the Fifty-sixth WHO Regional Committee for Africa, it might be helpful for Botswana to consider institutionalizing efficiency monitoring of health facilities within health management information systems.

## Background

Botswana is situated in Southern Africa and has a population of 1.921 million people - 61.1 percent of whom live in urban areas [1]. In 2008 the country generated total gross national income (GNI) of US$12,754 million and GNI per capita of US$6,640. In 2007 the country had a human development index (HDI) of 0.694, higher than the average HDI for Africa which stood at 0.547. Adult literacy stood at 82.9 percent and combined gross enrolment in education was 70.6 percent - higher than Africa's averages of 63.3percent and 55.9 percent respectively [2].

As can be seen in Table 1, health Millennium Development Goals (MDGs) indicators for Botswana are generally better than those for the African Region as a whole. The country also has a higher density of physicians, nursing and midwifery personnel, other health service providers and hospital beds than the average for the region, while health service coverage for indicators such as care of women during pregnancy and childbirth, reproductive health services, immunization to prevent common childhood infections, and treatment for HIV and Tuberculosis is also above regional averages. However, the prevalence of HIV among adults, at 22,757 per 100 000 population, is five times higher than the average for the region [3], while the neonatal mortality rate, adult mortality rate, HIV/AIDS -specific mortality rate, TB among HIV-positive people, incidence and prevalence of tuberculosis are all significantly higher than regional averages.

**Table 1:** Millennium Development Goals, health and national health accounts indicators

	Botswana	African Region
**Health-related Millennium Development Goals Indicators**		
Low birth weight newborns (percent)	10	14
Children aged <5 years underweight for age (percent)	10.7	21.3
Under-5 mortality rate (probability of dying by age 5 per 1000 live births	40	145
Measles immunization coverage among 1-year-olds (percent)	90	74
Maternal mortality ratio (per 100 000 live births)	380	900
Births attended by skilled health personnel (percent)	94	46
Contraceptive prevalence (percent)	44.4	24.4
Adolescent fertility rate (per 1000 girls aged 15-19 years)	51	117
Antenatal care coverage (percent): at least 1 visit	97	73
Prevalence of HIV among adults aged > = 15 years per 100 000 population	22757	4735
Antiretroviral therapy coverage among people with advanced HIV infection (percent)	79	30
Malaria mortality rate per 100 000 population	2	104
Tuberculosis treatment success under DOTS (percent)	72	75
Access to improved drinking-water sources (percent)	96	59
Access to improved sanitation (percent)	47	33
		
**Global Health Indicators**		
Life expectancy at birth in years	56	52
Healthy life expectancy (HALE) at birth (years)	49	45
Neonatal mortality rate (per 1000 live births)	46	40
Infant mortality rate (probability of dying between birth and age 1 per 1000 live births)	32	88
Adult mortality rate (probability of dying between 15 and 60 years per 1000 population)	514	401
HIV/AIDS -specific mortality rate (per 100 000 population)	585	198
Malaria -specific mortality rate (per 100 000 population)	2	104
TB among HIV-negative people (per 100 000 population)	37	45
TB among HIV-positive people (per 100 000 population)	156.5	47.6
Age-standardized mortality rates from non-communicable diseases (per 100 000 population)	594	841
Prevalence of tuberculosis (per 100 000 population)	622	475
Incidence of tuberculosis (per 100 000 population per year)	731	363
Prevalence of HIV among adults aged >15 years (per 100000 population)	22757	4735
		
**Health service coverage**		
Antenatal care coverage (percent) - at least 1 visit	97	73
Antenatal care coverage (percent) - at least 4 visit	97	45
Births attended by skilled health personnel (percent)	94	46
Births by caesarean section (percent)	7.7	3.3
Neonates protected at birth against neonatal tetanus (percent)	78	31
Immunization coverage among 1-year-olds (percent) - MDG 4 Measles	90	74
Immunization coverage among 1-year-olds (percent) - MDG 4 DPT3	97	74
Immunization coverage among 1-year-olds (percent) - MDG 4 HepB3	85	69
Antiretroviral therapy coverage (percent) - Pregnant women (PMTCT)	95	34
Antiretroviral therapy coverage (percent) - People with advanced HIV infection	79	30
Tuberculosis detection rate under DOTS (percent)	57	47
Tuberculosis treatment success under DOTS (percent)	72	75
		
**Health workforce and infrastructure**		
Physicians	715	150708
Physicians - Density (per 10 000 population)	4	2
Nursing and midwifery personnel	4753	792361
Nursing and midwifery personnel (per 10 000 population)	27	11
Dentistry personnel	38	23964
Dentistry personnel (per 10 000 population)	<1	1
Other health service providers	1611	257520
Other health service providers (per 10 000 population)	9	4
Hospital beds (per 10000 population)	24	10
		
**Health expenditure (2008)**		
Total expenditure on health as percent of Gross domestic product	5.6	5.7
General government expenditure on health as percent of total expenditure on health	74.3	50.5
Private expenditure on health as percent of total expenditure on health	25.7	49.5
General government expenditure on health aspercent of total government expenditure	11.7	10.1
External resources for health as percent of total expenditure on health	5.7	20.8
Social Security expenditure on health as percent General government expenditure on health	0.0	3.4
Out-of-Pocket expenditure as percent of private expenditure on health	27.3	73.3
Private prepaid plans as percent of private expenditure on health	5.2	7.1
Per capita total expenditure on health at average exchange rate (US$)	392.5	101.8
Per capita total expenditure on health (PPP int.$)	779.3	172.1
Per capita government expenditure on health at average exchange rate (US$)	291.5	59.8
Per capita government expenditure on health (PPP int.$)	578.8	99.8

Source: WHO [3].

In 2008, Botswana spent 5.6 percent of its gross domestic product (GDP) on health, which translated to a per capita total expenditure on health at purchasing power parity (PPP) of int$762 [4]. About 74.3 percent of total expenditure on health came from general government spending (i.e. approximately int$568 per person), the remainder coming from private spending. The latter comprises 27.35 percent of out-of-pocket spending by households and 5.2 percent from private prepaid plans. Botswana received only 5.7 percent of total expenditure on health from external sources.

In 2008 Botswana's under-5 mortality rate stood at 40 per 1000 live births, maternal mortality stood at 380 per 100000 live births, and life expectancy was 56 years [3]. This compares with under-5 mortality of 37 per 1000 live births, maternal mortality of 180 per 100000 live births, and life expectancy of 71 years in Algeria where total per capita expenditure on health is just int$338 (roughly half of Botswana's). Cape Verde also did more with less, per capita total expenditure on health of int$148, delivering an under-5 mortality rate of 32 per 1000 live births, maternal mortality of 210 per 100000 live births, and life expectancy of 70 years. Meanwhile Mauritius, with total per capita expenditure on health of int$502, achieved an under-5 mortality rate of 17 per 1000 live births, maternal mortality of 15 per 100000 live births, and life expectancy of 73 years. Algeria, Cape Verde and Mauritius all deliver better health outcomes in these areas at lower levels of per capita expenditure than Botswana.

Even though the majority of health sector resources in Botswana are spent on hospitals, to date no study has been attempted to address the following questions:

(a)	Are hospitals producing the maximum outputs with the available inputs?

(b)	Are hospitals operating at optimal scale?

(c)	What is the trend of hospital productivity?

(d)	What percentage of observed productivity changes can be attributed to technical efficiency change and technological change/growth?

The specific objectives of this study were to measure the technical and scale efficiency of hospitals in Botswana, and to evaluate changes in productivity over a three year period in order to analyze changes in efficiency and changes in technology. Finally the study seeks to highlight possible implications for government policy.

One of the policy statements under the Leadership and Governance rubric in Botswana's national health policy [5] reads: "The MOH shall from time to time review and revise its organisation and management structures to respond to new developments and challenges in order to gain and maintain high efficiency in the provision of health care (p. 18)". The government clearly views assuring efficiency in the health sector as one its key leadership and governance responsibilities. This study provides evidence that might help Botswana's policy-makers achieve their stated goals.

### Prior Research on Hospital Productivity

After an extensive literature search, Emrouznejad *et al *[6] identified more than 4000 research articles published in journals or book chapters applying Data Envelopment Analysis (DEA) to measure efficiency and productivity around the world. They found that banking, education (including higher education), health care, and hospital efficiency were the most popular application areas. O'Neill *et al *[7] did a systematic review of 79 DEA-based hospital efficiency studies published from 1984 to 2004 from 12 countries around the world. In the African Region, DEA has only been applied to analyse the efficiency of hospitals in Angola [8], Benin [9], Ghana [10], Kenya [11], Namibia [12], South Africa [13,14], Uganda [15] and Zambia [16]. The literature reviews by both Emrouznejad *et al *[6] and O'Neill *et al *[7] has shown that many studies have applied DEA to estimate efficiency of hospitals but that only a few addressed productivity growth.

In this section, we review a few recent studies that have used Malmquist DEA to decompose productivity of hospitals. Zere et al [14] used Mamquist DEA to assess changes in productivity from 1992/93 to 1997/98 in 10 acute care hospitals in the Western Cape Province of South Africa. The hospitals were assumed to use recurrent expenditures and beds to produce outpatient visits and inpatient days. The mean Malmquist total factor productivity change (MTFP) score was 0.879, efficiency change (EFFCH) score was 0.984, and technical change (TECH) score was 0.893. The decrease in Western Cape Province hospitals productivity was attributed to 1.6% regress in efficiency and 10.7% decline in technological innovation.

Ouellete and Vierstraete [17] evaluated productivity changes of emergency units of 15 hospitals in Montreal Canada for 1997-98 and 1998-99 using Malmquist DEA. The inputs used included non-physician hours worked, expenditure on furniture and equipment, number of stretchers, and full time equivalent number of physicians. The output was number of cases. The study found that overall mean MTFP was 0.92, EFFCH was 0.94 and TECH was 1.05. The 8% decrease in productivity was primarily attributed to a decrease in efficiency.

Barros *et al *[18] assessed productivity change of 51 hospitals in Portugal using both the Luenberger indicators and the Malmquist index during the years 1997 to 2004. The inputs included number of beds, number of full-time equivalent personnel, and total variable costs. The outputs included case flows (number of persons that leave the hospital), length of stay, number of consultations, and number of emergency cases. The Luenberger indicator was 0.008, EFFCH was -0.001 and TECH was 0.009. On the other hand, the MTFP was 1.042, EFFCH was 1.036 and TECH was 0.995. The Malmquist DEA results imply that on average productivity of the hospitals under consideration grew mainly due to improvement in efficiency.

Gannon [19] applied Malmquist DEA on samples of 6 regional, 8 general and 22 country hospitals in Ireland for the period 1995 to 1998. The inputs used were number of beds and full-time equivalent (FTE) people employed. The outputs included number of discharges and deaths, outpatient attendances, and day cases. The study revealed that regional hospitals had MTFP of 1.028, EFFCH of 0.994, and TECH of 1.034. The general hospitals had EFFCH equal to 0.999, TECH equal to 1.013 and MTFP equal to 1.012. The country hospitals had EFFCH of 1.005, TECH of 0.992 and MTFP of 0.997. Therefore, on average the productivity of both regional and general hospitals improved while that of county hospitals declined between 1995 and 1998.

Dash [20] applied Malmquist DEA to study productivity of 29 district headquarters hospitals in India during the years 2002 to 2007. The inputs included number of beds, number of nursing staff, and number of physicians (Surgeon). The outputs were number of inpatients, number of outpatients, number of surgeries undertaken, emergency cases handled, medico legal cases, and deliveries. The hospitals had a MTFP value of 1.2358, EFFCH of 1.15, and TECH of 1.07. Therefore, the 23.6% hospital productivity growth was explained by a 15% improvement in efficiency combined with a 7% increase in innovation.

NG [21] employed Malmquist DEA to assess productivity of 12 coastal hospitals and 17 inland hospitals in China from 2002 to 2005. The study used three inputs, i.e. number of doctors and nurses, other health staff, and beds; and two outputs, i.e. number of outpatient visits and inpatient stays. The coastal hospitals had a MTFP score of 1.1307, TECH score of 1.1467, and EFFCH score of 0.9860. The latter was attributed to a 1.41% increase in SECH and a 2.77% regress in pure efficiency change. Contrastingly, inland hospitals MTFP was 0.9853; which was explained by both a TECH score of 1.0851 and an EFFCH score of 0.9080. The 9.2% efficiency regress was attributed to deterioration in both scale efficiency change (SECH) of 3.57% and pure technical efficiency change (PECH) of 5.83%.

Kirigia *et al *[8] estimated the performance of 28 municipal hospitals in Angola using Malmquist DEA during the period 2000-2002. The inputs included were number of physicians plus nurses, number of beds, and expenditures on pharmaceutical and non-pharmaceutical supplies. The hospitals were assumed to produce two outputs, i.e. number of OPD visits and inpatient admissions. The municipal hospitals productivity grew by 4.5% (MTFP = 1.045). That growth was attributed to an average efficiency improvement of 12.7% (EFFCH = 1.127) and a technological regress of 7.3% (TECH = 0.927). The improvement in EFFCH was due to a 5% increase in PECH (PECH = 1.050) and a 7.3% increase in SECH (SECH = 1.073).

Dimas *et al *[22] used Malmquist DEA to examine the efficiency and productivity of 22 Greek public general hospitals for the period 2003-2005. The inputs employed included number of beds, total personnel salary, and total expenditure on medicines, supplies and other materials. The outputs included number of patient-days, number of patients in the outpatient department, and number of emergency cases. In 2003/2004 the MTFP was 1.02, depicting a 2% productivity growth; which was attributed to a 5% increase in innovation (TECH = 1.05) and a 1% decline in efficiency (EFFCH = 0.99). The latter was due to PECH of 0.97 and SECH of 1.02. In 2003/2005 hospital productivity declined by 5% (MTFP = 0.95) due to decrease in efficiency of 4% (EFFCH = 0.96) tempered by a 2% increase in innovation (TECH = 1.02). The efficiency regress was attributed to PECH of 0.98 and SECH of 0.99.

Karagiannis and Velentzas [23] applied Malmquist DEA to estimate productivity changes after the inclusion of quality variables for a panel of 8 Greek public hospitals during the period 2002-2007. The inputs included number of beds, the number of doctors, and the number of nursing and other personnel. The output used was number of inpatient days. The authors did estimations of the model excluding (Model A) and including (Model B) the quality variable. According to the conventional Malmquist productivity index (Model A), productivity decreased with an average of 1.2% during the period 2002-2007 (MTFP = 0.988). There occurred technical regress of 0.4% (TECH = 0.996) and an efficiency deterioration of 0.9% (EFFCH = 0.991). The latter resulted from a 1.3% (PECH = 0.987) deterioration in PECH tempered with a 0.4% (SECH = 1.004) increase in SECH. According to the quality adjusted Malmquist productivity index (Model B), productivity regressed by 1.4% (MTFP = 0.986) due to a 0.3% regress in innovation (TECH = 0.997) and a 1.2% decrease in efficiency (EFFCH = 0.988). There occurred a 0.2% (QCH = 1.002) increase in overall productivity due to improvement in quality.

Lobo *et al *[24] evaluated the performance and productivity change among 30 Brazilian Federal University hospitals during the years 2003 to 2006 using Malmquist DEA. The inputs used included labor force (physicians and full time equivalent non-physicians), operational expenses (not including payroll), beds, and service-mix. Whilst, the outputs included number of admissions, inpatient surgeries and outpatient visits. The University hospitals productivity growth of 20.9% (MTFP = 1.20859) was attributed solely to increase of 36.5% in innovation (TECH = 1.36542) tempered by a 10.3% decrease in efficiency (EFFCH = 0.89716).

Chang *et al *[25] examined the impact of Taiwan Quality Indicator Project (TQIP) participation on 31 regional hospitals productivity over the periods 1998-2002 and 1998-2004 using Malmquist DEA. The inputs utilized included number of physicians, nurses, supporting ancillary services personnel, and patient beds. The hospitals were assumed to produce number of outpatient visits, ambulatory and emergency room visits, patient days, and net inpatient mortalities. In 1998-2002 the MTFP was 0.7877, quality change (QC) was 0.8645, EFFCH was 0.9712 and TECH was 0.9520. In 1998-2004 MTFP was 0.8748, QCH was 0.8939, EFFCH was 0.9792 and TECH was 0.9873.

## Methods

A health system includes all organizations, institutions and activities whose primary purpose is to promote, restore or maintain people's health, i.e. both length of life and health-related quality of life [26]. One of the pivotal health system institutions is the hospital which combines limited health system inputs (e.g. health workforce, medical products, non-medical supplies, clinical technologies, beds, building space, ambulances) to produce preventive, curative and rehabilitative services.

In the context of hospitals, technical efficiency means making the best use of given quantities of health system inputs and existing technology. Allocative efficiency on the other hand is relates to the optimal allocation of health system inputs. Economic efficiency is a product of both technical efficiency and allocative efficiency [27]. Our study focuses on the measurement of technical efficiency using Data Envelopment Analysis (DEA).

### Data Envelopment Analysis (DEA)

DEA is a linear programming method designed to measure the relative efficiencies of a set of Decision-Making Units (DMU) such as hospitals [6,7]. DEA measures the efficiency of a DMU relative to the efficiency of its peer group, with a notional 'production frontier' representing optimal efficiency. All DMUs lay on or below the 'production frontier'. DEA solves as many linear programming problems as the number of the DMUs in the study sample [28].

The efficiency of a hypothetical hospital producing one health service output from one health system input would be obtained by dividing the quantity of that output by the quantity of the input. However, 'real world' hospitals use multiple inputs (e.g. health workforce, medicines, non-medical supplies, capital inputs) to produce multiple outputs (e.g. preventive, curative, rehabilitative services). In such a scenario, the efficiency of a hospital needs to be expressed as the weighted sum of health service outputs divided by the weighted sum of health system inputs.

According to Charness *et al *[28], efficiency (E) of a target hospital from the set "j" can then be obtained by solving the following fractional programming model:

(1) $$\begin{array}{l} Max\;\;E_{0}=\sum_{R=1}^{s}U_{r}Y_{r0}/\sum_{I=1}^{m}V_{i}X_{i0}\\ Subject\;\;to:\;E_{j}\leq 1,\;j=1,..,n\\ u_{r},v_{r}\geq 0 \end{array}$$

where: *Y*_*rj *_is the amount of health service output *r *(*r *= 1,..., *s*) from hospital *j*; *X*_*ij *_is the amount of health system input *j *(*i = *1,..., *m*)in *j*^*th*^hospital; *u*_*r *_is the weight given to health service output *r*; *v*_*i *_is a weight given to health system input *i*; and *n *is the number of hospitals in the sample.

Charnes *et al *[28] converted model (1) into the following constant returns to scale (CRS) linear programming model:

(2) $$\begin{array}{l} Max\;\;E_{0}\;\;=\;\;\sum u_{r}y_{rj0}=1\\ subject\;\;to:\;\;\sum_{r=1}^{s}v_{i}x_{ij0}=1\\ \sum_{i=1}^{m}u_{r}y_{rj}\;-\;\sum v_{i}x_{ij}\leq 0,\;\;j=1,...,N. \end{array}$$

The latter constraint means that all DMU's are either on or below the frontier. Model (2) implies that increases in the amount of health system inputs will be matched by increases in outputs, e.g. the doubling of inputs leads to a doubling of outputs. This CRS model assumes that hospitals are operating at an optimal scale of production, and hence, technical efficiency is equal to scale efficiency.

However, in reality a hospital can manifest either CRS, increasing returns to scale (IRS) or decreasing returns to scale (DRS). In an IRS (or economies of scale) scenario, increases in the amount of health system inputs will result in a proportionately greater increase in outputs, e.g. a doubling of all inputs will lead to more than a doubling of outputs. Where a hospital is experiencing DRS (diseconomies of scale), a doubling of inputs would lead to less than a doubling of outputs. In order to allow for the variability of returns to scale, the linear programming problem (3) was estimated for each hospital in the sample [29]:

(3) $$\begin{array}{l} Max\;\;E_{0}\;\;=\;\;\sum u_{r}y_{rj0}\;\;+\;\;U_{0}\\ s.t.:\;\;\sum_{r=1}^{s}v_{i}x_{ij0}=1\\ \sum_{i=1}^{m}u_{r}y_{rj}\;-\;\sum v_{i}x_{ij}\;+\;U_{0}\leq 0,\;\;j=1,...,N\\ U_{r},V_{i}\geq 0. \end{array}$$

The relative efficiency score (E) lies between 0 (totally inefficient), and 1 (optimal technical efficiency).

### Malmquist Productivity Index

Productivity is the measure of the relationship between the outputs of a hospital and the health system inputs that have gone into producing those outputs. An increase in productivity occurs when output per health worker hour is raised and/or there is use of more and/or better health technology. In general a productivity index is defined as the ratio of an output quantity index to an input quantity index, i.e.:

(4) $$P_{t}=\frac{Y_{t}}{X_{t}};$$

Where: *P*_*t *_is a productivity index; time period *t *= 0,...,*T*; *Y*_*t *_is an output quantity index and *X*_*t *_is an input quantity index. Each index represents accumulated growth from period 0 to period *t*. In a real hospital, *X*_*t *_comprise more than one input and *Y*_*t *_more than one output. Because hospital outputs and inputs are heterogeneous it is not possible to add all outputs to form an output quantity index or to add all inputs to form an input quantity index. Disaggregated data on the quantities of outputs and inputs need weighting to form output and input quantity indices [30].

We opted to use the DEA-based Malmquist Productivity Index (MPI) to study efficiency and productivity changes over a period of time for a number of reasons: it requires information solely on quantities of inputs and outputs and not on their prices; it does not require the imposition of functional form on the structure of production technology; it easily accommodates multiple hospital inputs and outputs; and it can be broken down into the constituent sources of productivity change - i.e. efficiency changes and technological changes [31]. An increase in the efficiency level can be interpreted as a move by the hospital to 'catch-up' with the efficiency frontier. Improvement in hospital's health technology shifts the efficiency frontier upward [27].

Malmquist DEA is applied to panel data to calculate indices of changes in Total Factor Productivity (TFP), technology, technical efficiency and scale efficiency. The MPI takes a value of more than one for productivity growth, a value of one for stagnation and a value of less than one for productivity decline. The output-oriented MPI is defined as the geometric mean of two periods' productivity indices, subsequently broken down into various sources of productivity change [32]:

(5) Mo(yt+1,xt+1,yt,xt)=[D0t+1(xt+1,yt+1)D0t(xt,yt)]×︸EFFCH[D0t(xt+1,yt+1)D0t+1(xt+1,yt+1)×D0t(xt,yt)D0t+1(xt,yt)]12︸TECH

Where *D *represents the distance function; the *M*_0 _is the output oriented Malmquist productivity index; EFFCH is change in relative hospital efficiency (i.e., the change in the gap between observed production and maximum feasible production) between years *t *and *t *+ 1; TECH is a measure of the technical change in the hospital production technology, i.e. it measures the shift in technology use between years *t *and *t *+ 1.

EFFCH estimated under the assumption of CRS can be broken down to allow identification of change in scale efficiency, i.e. the productivity change resulting from a scale change that brings the hospital closer to or farther away from the optimal scale of output as identified by a VRS technology [32]:

(6) $$\begin{array}{l} \underset{CRS\;\;\;EFFCH}{\underset{︸}{\left[\frac{D_{0}^{t+1}{\left(x^{t+1},y^{t+1}\right)}_{CRS}}{D_{0}^{t}{\left(x^{t},y^{t}\right)}_{CRS}}\right]}}=\underset{Pure\;\;EFFCH}{\underset{︸}{\frac{D_{0}^{t+1}{\left(x^{t+1},y^{t+1}\right)}_{VRS}}{D_{0}^{t}{\left(x^{t},y^{t}\right)}_{VRS}}}}\times \\ \underset{Scale\;\;EFFCH}{\underset{︸}{\left[\frac{D_{0}^{t}{\left(x^{t},y^{t}\right)}_{VRS}}{D_{0}^{t}{\left(x^{t},y^{t}\right)}_{CRS}}\times \frac{D_{0}^{t+1}{\left(x^{t+1},y^{t+1}\right)}_{CRS}}{D_{0}^{t+1}{\left(x^{t+1},y^{t+1}\right)}_{VRS}}\right]}}\; \end{array}$$

Where CRS (or VRS) signify a gap measured under the assumption of constant (or variable) returns-to-scale. Pure EFFCH (the first term on the right) measures change in technical efficiency under the assumption of a VRS technology. Scale EFFCH (the bracketed term on the right) in a given period captures the deviations between the VRS technology and the CRS technology at observed inputs, i.e. it measures changes in efficiency due to movement toward or away from the point of optimal hospital scale.

The *M*_0 _attains a value greater than, equal to, or less than one if a hospital has experienced productivity growth, stagnation or productivity decline, net of the contribution of scale economies, between periods t and t+1. EFFCH is greater than one, equal to or less than one if a hospital is moving closer to, unchanging or diverging from the production frontier. TECH is greater than, equal to or less than one when the technological best practice is improving, unchanged, or deteriorating, respectively [33].

### Data

Botswana's health system consists of 3 referral hospitals under the Ministry of Health (MOH), 7 district hospitals also under the MOH, 2 mission district hospitals (fully funded by government), 3 mine hospitals, 2 private hospitals, 17 primary hospitals under MOH control and an array of private general practitioners. There are also 104 health clinics with beds, 173 health clinics without beds, 349 health posts and 856 mobile posts under the Ministry of Local Government. Both district and primary hospitals refer patients to the three referral hospitals - Princess Marina in Gaborone, Nyangabgwe Hospital in Francistown and Lobatse Mental Hospital [34]. District and primary hospitals were the focus of this study. A simple random sample of 21 (67.7 percent) hospitals was drawn from the 31 district and primary hospitals and included Kanye and Bamalete mission hospitals as well as the Delta private hospital.

The data used in this study covers the period from 2006 to 2008 and was collected through visits to all the 21 primary and secondary hospitals in Botswana in 2009 by JN and NT using the WHO/AFRO hospital efficiency questionnaire [35]. The study is based on two inputs: (i) the number of clinical staff (physicians, nursing and midwifery personnel, dentistry personnel, other technical health service providers); and (ii) the number of hospital beds as a proxy of capital inputs. Due to the absence of disaggregated data, we omitted the cost of medical products (medicines, vaccines and technologies) and non-medical supplies.

According to English *et al *[36], beyond offering outpatient and inpatient medical and surgical services, district hospitals also play important roles in health-related information, communication, coordination, and training, including: integration with other local health-related services, such as water and sanitation; training of health workers; supervision and monitoring of health workers in the peripheral health centres; and managing health information systems. Although we were cognizant of the latter four services, it was not possible to factor them in, due to the limited scope of our study. This study used only two outputs: (i) number of outpatient department visits, and (ii) number of inpatient days. The choice of inputs and outputs was based on the published hospital efficiency literature in the African Region [12,14]. We were not able to verify whether all the hospitals had exactly the same standards in terms of type of services provided, quality of care provided, qualification and experience of staff, working schedules, functional building capacity, hospital technology, etc.

DEAP computer program [37] was used in the estimation of the yearly hospital efficiencies and the Malmquist Productivity Indices.

## Results and Discussion

### Technical Efficiency

Table 2 presents individual hospitals' technical and scale efficiency scores during the three years. In 2006, 2007 and 2008 out of the 21 hospitals:

**Table 2:** Hospital's technical and scale efficiency during 2006-2008

Hospitals	Efficiency 2006				Efficiency 2007				Efficiency 2008			
	
	CRSTE	VRSTE	SCALE	Returns to Scale	CRSTE	VRSTE	SCALE	Returns to Scale	CRSTE	VRSTE	SCALE	Returns to Scale
Rakops	0.511	0.54	0.946	DRS	0.559	0.615	0.909	IRS	0.552	0.587	0.94	IRS

Masunga	0.277	0.315	0.879	DRS	0.263	0.272	0.964	IRS	0.272	0.278	0.978	IRS

Mmadinare	0.536	0.557	0.962	IRS	0.531	0.54	0.984	IRS	0.473	0.484	0.977	IRS

Bobonong	1	1	1	CRS	1	1	1	CRS	1	1	1	CRS

Palapye	0.834	0.947	0.881	DRS	0.651	0.772	0.844	DRS	0.489	0.633	0.774	DRS

Letlhakane	1	1	1	CRS	1	1	1	CRS	1	1	1	CRS

Sefhare	0.49	0.522	0.939	DRS	0.454	0.485	0.936	IRS	0.444	0.463	0.958	IRS

Thebephatswa	0.352	0.371	0.948	IRS	0.403	0.426	0.946	IRS	0.478	0.514	0.931	IRS

Gweta	0.352	0.37	0.952	DRS	0.257	0.261	0.986	IRS	0.347	0.363	0.955	IRS

Kanye	0.79	1	0.79	DRS	0.973	1	0.973	DRS	0.931	1	0.931	DRS

Orapa	0.467	1	0.467	DRS	0.636	0.991	0.642	DRS	0.619	0.993	0.624	DRS

Maun	0.336	0.463	0.727	DRS	0.354	0.591	0.599	DRS	0.354	0.827	0.428	DRS

Phikwe	0.712	0.93	0.766	DRS	0.7	0.905	0.773	DRS	0.643	1	0.643	DRS

Scottish	0.365	0.506	0.723	DRS	0.513	0.836	0.613	DRS	0.385	0.881	0.437	DRS

Sekgoma	0.394	0.748	0.527	DRS	0.323	0.883	0.366	DRS	0.413	1	0.413	DRS

Athlone	0.409	0.512	0.798	DRS	0.634	0.642	0.988	IRS	0.387	0.59	0.657	DRS

Mahalapye	0.672	0.854	0.788	DRS	0.636	0.965	0.659	DRS	0.525	1	0.525	DRS

Delta	0.447	1	0.447	IRS	0.848	1	0.848	IRS	0.785	1	0.785	IRS

Jwaneng	0.441	0.727	0.606	DRS	0.803	0.809	0.994	IRS	0.622	0.624	0.996	IRS

Bamalete	0.638	0.862	0.74	DRS	1	1	1	CRS	1	1	1	CRS

DEBORAH	0.416	0.563	0.739	DRS	0.45	0.596	0.755	DRS	0.482	0.791	0.61	DRS

**MEDIAN**	**0.467**	**0.727**	**0.79**		**0.634**	**0.809**	**0.936**		**0.489**	**0.827**	**0.931**	

**MEAN**	**0.467**	**0.704**	**0.792**		**0.618**	**0.742**	**0.847**		**0.581**	**0.763**	**0.789**	

**STDEV**	**0.215**	**0.246**	**0.169**		**0.245**	**0.248**	**0.179**		**0.231**	**0.249**	**0.216**	

■ Five (24 percent), five (24 percent) and eight (38 percent) hospitals registered a variable returns to scale technical efficiency (VRSTE) score of 100 percent, respectively.

■ Therefore, 16 (76 percent), 16 (76 percent) and 13 (62 percent) of the hospitals can be said to have been run inefficiently over the same time period.

Average VRSTE scores of Botswana stood at 70.4 percent, 74.2 percent and 76.3 percent. This finding implies that if run efficiently, the hospitals could have produced 29.6 percent, 25.8 percent and 23.7 percent more output (number of outpatient department visits and number of inpatient days) for the same volume of inputs.

These average VRSTE scores were higher than those of Angola (65.8% - 67.5%) [8], Ghana (61%) [10] and Zambia (67%) [16]. However, they were fairly similar to those obtained in Benin (63.3% - 85.8%) [9], Kenya (84%) [11], Namibia (62.7% - 74.3%) [12], and Eastern, Northern and Western Cape Provinces of South Africa (82% - 82.8%) [14]. Technical efficiency scores for Kwazulu-Natal Province of South Africa (90.6%) [13] and Uganda (90.2% - 97.3%) [15] were significantly higher than those of Botswana.

### Scale Efficiency

In 2006, 2007 and 2008, out of the 21 hospitals:

■ Two (9.5 percent), three (14.3 percent) and three (14.3 percent) hospitals showed constant returns to scale (CRS). Among these hospitals, the doubling of health system inputs lead to a doubling of health service outputs. In other words the size of these hospitals did not affect productivity. The average and marginal productivity of these hospitals remained constant whether the hospital was small or large [38]. In short, they were operating at their most productive scale.

■ Three (14.3 percent), nine (42.9 percent) and eight (38.1 percent) hospitals manifested increasing returns to scale (IRS). This may have arisen because the larger scale of a particular operation allowed health managers and workers to specialize in their tasks and make use of more sophisticated health technologies [38]. Hospitals manifesting IRS ought to expand their scale of operation in order to become scale efficient.

■ Sixteen (76.2 percent), 9 (42.9 percent) and 10 (47.6 percent) hospitals experienced decreasing returns to scale (DRS) which may be associated with the problems of coordinating tasks and maintaining lines of communication between management and workers [38]. The hospitals experiencing DRS need to reduce their scale of operation in order to operate at the most productive scale size [39].

The average scale efficiency score was 79.2 percent in 2006, 84.7 percent in 2007 and 78.9 percent in 2008, indicating that there is scope to increase total hospital outputs by approximately 20.9 percent in 2006, 15.3 percent in 2007 and 21.1 percent in 2008. Where technically and politically feasible this can be achieved through the appropriate reduction in the size of the scale-inefficient hospitals.

These average scale efficiency scores were within the range of those for Angola (81%-89%) [8], Benin (41.9%-73.6%) [9], Ghana (81%) [10], Namibia (73.2%-83.7%) [12], South Africa (Eastern, Northern and Western Cape Provinces) (82.5%-90%) [14] and Zambia (80%) [16]. However, the average scale efficiency scores for Botswana were lower that those of Kenya (90%) [11], Kwazulu-Natal Province of South Africa (95.3%) [13], and Uganda (97.5%) [15].

### Scope for output increases (input reductions) and implications for policy

Table 3 shows the output (input) increases (reductions) needed to make individual inefficient hospitals efficient during the three years. Table 4 reports the total output increases and/or input reductions needed to make inefficient hospitals efficient. In 2008, for example, the inefficient hospitals combined would need to increase outpatient visits by 117,627 (18 percent) and inpatient days by 49,415 (13 percent) so as to become efficient. Alternatively, the inefficient hospitals could become efficient by reducing the number of clinical staff by 264 (9 percent) and number of beds by 39 (2 percent).

**Table 3:** Output (Input) increases (reductions) needed to make individual inefficient hospitals efficient during 2006-2008

Hospital	Outputs 2006		Inputs 2006		Outputs 2007		Inputs 2007		Outputs 2008		Inputs 2008	
	
	Outpatient visits	Inpatient days	Outpatient visits	Inpatient days	Outpatient visits	Inpatient days	Outpatient visits	Inpatient days	Outpatient visits	Inpatient days	Outpatient visits	Inpatient days
Rakops	16,029	0	25	0	19,373	0	0	0	23,765	0	0	0

Masunga	16,807	0	2	0	19,413	0	0	0	17,150	0	0	0

Mmadinare	0	4,887	0	24	0	12,872	0	0	0	14,345	0	0

Bobonong	0	0	0	0	0	0	0	0	0	0	0	0

Palapye	10,333	0	90	0	10,419	0	31	0	745	0	0	0

Letlhakane	0		0	0	0	0	0	0	0	0	0	0

Sefhare	11,509	0	6	0	14,354	0	0	0	13,220	0	0	0

Thebephatswa	0	7,370	0	15	0	7,779	0	0	0	10,955	0	0

Gweta	12,518	0	0	16	0	2,350	0	0	5311	0	0	0

Kanye	0	0	0	0	0	0	0	0	0	0	0	0

Orapa	0	0	0	0	0	12,732	54	0	0	14,467	60	0

Maun	35,969	0	51	0	4,186	0	165	0	0	0	90	11

Phikwe	38,844	0	103	0	46,590	0	77	0	0	0	0	0

Scottish	25,197	0	42	0	49,543	0	127	0	7,792	0	0	27

Sekgoma	64,196	0	94	183	55,601	0	160	183	0	0	0	0

Athlone	28,688	0	0	17	27,187	0	0	43	26,357	0	67	0

Mahalapye	33,365	0	174	0	42,660	0	143	0	0	0	0	0

Delta	0	0	0	0	0	0	0	0	0	0	0	0

Jwaneng	0	1,073	0	25	0	13,579	0	0	0	9,648	0	0

Bamalete	2,728	0	66	0	0	0	0	0	0	0	0	0

Deborah	29,739	0	25	0	33,009	0	98	0	23,287	0	47	0

**TOTAL**	**325,922**	**13,330**	**678**	**280**	**322,336**	**49,312**	**857**	**226**	**117,627**	**49,415**	**264**	**39**

**MEDIAN**	**11,509**	**0**	**2**	**0**	**4,186**	**0**	**0**	**0**	**0**	**0**	**0**	**0**

**MEAN**	**15,520**	**667**	**32**	**13**	**15,349**	**2,348**	**41**	**11**	**5,601**	**2,353**	**13**	**2**

**STDEV**	**176,499**	**7,696**	**48**	**40**	**175,955**	**28,470**	**61**	**41**	**9,223**	**5,059**	**27**	**6**

**Table 4:** Total output (input) increases (reductions) needed to make inefficient hospitals efficient

	Total actual values (2006)	Shortfall/excess (2006)	Total actual values (2007)	Shortfall/excess (2007)	Total actual values (2008)	Shortfall/excess (2008)
OPD visits	626,350	325,922 (52percent)	652,401	322,336 (49percent)	648,185	117,627 (18 percent)

Inpatient days	368,985	13,330 (4percent)	350,057	49,312 (14percent)	383,597	49,415 (13 percent)

Clinical staff	2,803	678 (24percent)	2,599	857 (33percent)	2,956	264 (9 percent)

Beds	2,047	280 (14percent)	2,067	226 (11percent)	2,374	39 (2 percent)

With regard to hospitals with outputs falling short of the DEA targets, MOH policy-makers in Botswana could improve efficiency by improving access and utilization of under-utilized neonatal, infant and maternal health services, some of which are mentioned in Table 1. This may require a multi-pronged strategy involving:

■ Use of health promotion strategies and techniques such as: advocacy; social mobilization; social marketing; information, education and communication (IEC); regulation and legislation; partnerships and alliances with public, private, nongovernmental organizations and civil society; and inter-sectoral action to address determinants of health to improve the use of under-utilized health services [40].

■ Ensuring universal access to health services through pooled pre-paid contributions collected on the basis of ability to pay through either tax-based funding or social health insurance, or a mix of both [41-43]. This would be in line with Botswana's national health policy statement that [5]: "The Government shall ensure availability of financial resources for a prepaid package of essential health interventions to all people living in Botswana so that the services are available to the client free of charge" (p.23).

Alternatively, if it is not feasible to attenuate inefficiencies through the improved utilization of hospital health services, policy-makers could achieve the same objective through the transfer of excess clinical staff and beds to health clinics, health posts and mobile health posts. This course of action would need to be guided by an efficiency analysis of these lower level health facilities.

### Productivity growth

The year 2006 has been taken as the technology reference when using the Malmquist Total Factor Productivity (MTFP) index to analyze differences in productivity over time and it is worth remembering that according to the index, a value of less than one denotes deterioration in performance, values greater than one denote improvements in performance, and a value of one signifies no change.

Table 5 presents the Malmquist index summary of annual geometric means. In the last row (last column), we observe that, on average MTFP decreased slightly by 1.5 percent over the 2006-2008 period for the sample. On average, the deterioration in MTFP was due to technical change rather than efficiency change. Whereas hospitals efficiency increased by 3.5 percent, technical change (innovation) regressed by 4.5 percent. The efficiency change was attributed to an increase in pure efficiency of 4.2 percent and a decline in scale efficiency of 1 percent. MTFP change was the highest in 2008 (MTFP = 1.008) and the lowest in 2007 (MTFP = 0.963). The Botswana district hospitals average MTFP score of 0.985 was comparable to those obtained in Montreal Canada of 0.92 [17], China Inland of 0.9853 [21], Greece of 0.986-0.988 [23], Ireland County of 0.977 [19], South Africa of 0.879 [14] and Taiwan of 0.7877 [25]. Unlike Botswana, a number of countries hospitals had an average MTFP score greater than one signifying productivity growth: Angola municipal hospitals had 1.045 [8]; Brazilian Federal University hospitals had 1.209 [24]; China coastal hospitals had 1.1307 [21]; India district hospitals had 1.2358 [20]; Ireland regional hospitals had 1.028 [19]; and Portugal hospitals had 1.042 [18].

**Table 5:** Malmquist index summary of annual means (output oriented)

year	Efficiency change [A = (CxD]	Technical change [B]	Pure efficiency change [C]	Scale efficiency change [D = (A/C)]	Malmquist index of total factor productivity change [E = A*B]
2007	1.119	0.86	1.049	1.067	0.963
2008	0.95	1.061	1.035	0.918	1.008
**Mean**	**1.035**	**0.955**	**1.042**	**0.990**	**0.985**

Table 6 provides a summary of the annual geometric mean values of the MPI and its components for each hospital. Nine (42.9 percent) out of 21 hospitals had MPI scores greater than one, indicating growth in productivity. Rakops, Letlhakane, Kanye, Maun, Phikwe, Mahalpye, Delta, Bamalete and Deborah hospitals registered MTFP growth of 7.4 percent, 1.2 percent, 1.8 percent, 1.9 percent, 1.8 percent, 7.5 percent, 8.2 percent, 22.4 percent and 4.5 percent respectively. The productivity growth in Letlhakane, Phikwe and Mahalpye hospitals was attributed to technological innovation only. Meanwhile, productivity growth in hospital performance in the Kanye, Maun, Delta, Bamalete and Deborah hospitals was due to improvements in efficiency only. It was only in Rakops hospital where total factor productivity growth was fully explained by both improvements in efficiency and technological innovation.

**Table 6:** Malmquist index summary of firm means

	Efficiency change [A = (CxD]	Technical change [B]	Pure efficiency change [C]	Scale efficiency change [D = (A/C)]	Malmquist index of total factor productivity change [E = A*B]
Rakops	1.039	1.034	1.042	0.997	1.074
Masunga	0.991	0.95	0.94	1.054	0.942
Mmadinare	0.939	0.816	0.932	1.007	0.766
Bobonong	1	0.997	1	1	0.997
Palapye	0.766	1.198	0.817	0.937	0.918
Letlhakane	1	1.012	1	1	1.012
Sefhare	0.951	0.948	0.942	1.01	0.902
Thebephatswa	1.165	0.818	1.176	0.991	0.953
Gweta	0.992	0.912	0.99	1.002	0.905
Kanye	1.086	0.937	1	1.086	1.018
Orapa	1.152	0.845	0.997	1.156	0.973
Maun	1.026	0.993	1.337	0.768	1.019
Phikwe	0.95	1.071	1.037	0.917	1.018
Scottish	1.026	0.916	1.32	0.778	0.94
Sekgoma	1.024	0.975	1.156	0.885	0.998
Athlone	0.974	0.963	1.073	0.907	0.938
Mahalapye	0.884	1.217	1.082	0.816	1.075
Delta	1.325	0.816	1	1.325	1.082
Jwaneng	1.188	0.816	0.926	1.282	0.969
Bamalete	1.252	0.978	1.077	1.162	1.224
Deborah	1.077	0.97	1.185	0.909	1.045
**MEAN**	**1.031**	**0.955**	**1.042**	**0.99**	**0.985**

Note: All Malmquist index averages are geometric means

Conversely, 12 (57.1 percent) of the hospitals had Malmquist indices scores of less than one, indicating deterioration in productivity over time. Productivity regression in the Palapye hospital was solely due to a decline in efficiency. In Masunga, Mmadinare, Sefhare, Gweta and Athlone hospitals productivity regression was attributed to both declines in efficiency and innovation, whilst in Bobonong, Thebephatwa, Orapa, Scottish, Sekgoma and Jwaneng hospitals productivity regression was due solely to deterioration in technological innovation.

#### Pure efficiency change

As shown in Table 6, ten hospitals had an average pure efficiency change (PECH) score of greater than one. Hospitals registering a pure technical efficiency increase, included Phikwe hospital with 3.7 percent, 4.2 percent at Rakops, 7.3 percent at Athlone, Bamalete, with 7.7 percent, 8.2 percent at Mahalapye, 15.6 percent at Sekgoma, 17.6 percent at Thebephatswa, 18.5 percent at Deborah, 32 percent at Scottish and 33.7 percent at Maun.

Bobonong, Delta, Kanye and Letlhakane hospitals registered a PECH score of one, indicating no change in efficiency at those hospitals between 2006 and 2008. On the other hand, Palapye, Jwaneng, Mmadinare, Masunga, Sefhare, Gweta and Orapa experienced a decline in PECH of 18.3 percent, 7.4 percent, 6.8 percent, 6 percent, 5.8 percent, 1 percent and 0.3 percent respectively. The average PECH score for the entire sample was 1.042, implying that PECH reduced efficiency change by 4.2 percent.

#### Scale efficiency change

Scale efficiency change (SECH) is expressed as a value of less than, equal to, or greater than one if a hospital's scale of production contributes negatively, not at all, or positively to productivity change [20]. The scale of production in Gweta, Mmadinare, Sefhare, Masunga, Kanye, Orapa, Bamaleta, Jwaneng and Delta hospitals contributed positively to TFP change by a factor of 0.2 percent, 0.7 percent, 1 percent, 5.4 percent, 8.6 percent, 15.6 percent, 16.2 percent, 28.2 percent and 32.5 percent respectively.

Bobonong and Letlhakane hospitals had a scale index value of one, meaning that those hospitals scale of production did not contribute to MTFP change. On the other hand, the SECH score for 10 hospitals was less than one. This inidicates that the scale of production in Maun, Mahalapye, Sekgoma, Athlone, Deborah, Phikwe, Palapye, Thebephatswa and Rakops hospitals contributed negatively to productivity change by 23.2 percent, 22.2 percent, 18.4 percent, 11.5 percent, 9.3 percent, 9.1 percent, 8.3 percent, 6.3 percent, 0.9 percent and 0.3 percent respectively. The average SECH score for the entire sample was 0.99, indicating that the scale of production on average reduced efficiency change by 1 percent.

#### Technical Change

Sixteen hospitals (76.2 percent) registered technical change (TECH) of less than one, indicating a decline in technical innovation. The lack of technological innovation in Mmadinare, Delta, Jwaneng, Thebephatswa, Orapa, Gweta, Scottish, Kanye, Sefhare, Masunga, Athlone, Deborah, Sekgoma, Bamalete, Maun and Bobonong hospitals led to a decrease in TFP change of 18.4 percent, 18.4 percent, 18.4 percent, 18.2 percent, 15.5 percent, 8.8 percent, 8.4 percent, 6.3 percent, 5.2 percent, 5 percent, 3.7 percent, 3 percent, 2.5 percent, 2.2 percent, 0.7 percent and 0.3 percent respectively. Letlhakane, Rakops, Phikwe, Palapye and Mahalapye registered technological growth or progress between the periods *t *and *t *+ 1 of 1.2 percent, 3.4 percent, 7.1 percent, 19.8 percent and 21.7 percent respectively.

The technological progress showed by certain hospitals suggests that they applied an advance in scientific knowledge in the form of inventions or innovations with regard to both physical and human capital, such that it allowed a greater output and probably quality of health services, with health system input prices held constant. The advances may have resulted from the application of improved health technologies to health service production processes, but they may also have resulted from increases in health workforce motivation or skill and from improvements in health services organization.

Technological progress (or regression) depends on a number of factors, including: the availability of appropriate health technology (i.e. ecologically relevant and versatile for easy adaptation/application, requiring a minimum of new skills) plus complementary inputs and institutional changes; the existence of channels of communication between health policy-makers and hospital management teams; access to new appropriate technologies at affordable prices; availability of training facilities/opportunities to enable relevant health workforce to acquire new skills to take full advantage of a new technological possibility; and the availability of funds to finance the needed health technology investments [44].

## Conclusion

This study has measured the technical and scale efficiency of 21 hospitals in Botswana; quantified the output (input) increases (reductions) necessary to make inefficient hospitals efficient; and estimated the magnitude and sources of total factor productivity change within each hospital. The results indicate that 16 (76.2 percent), 16 (76.2 percent) and 13 (61.9 percent) of the 21 hospitals were run inefficiently between 2006, 2007 and 2008.

In 2008, the inefficient hospitals, taken together, would need to increase the number of outpatient visits by 117627 (18 percent) and inpatient days by 49415 (13 percent) in order to become efficient. There is scope for providing more child and maternal health services to additional persons by using the existing health system inputs more efficiently, i.e. without waste.

Alternatively, inefficiencies could be reduced by transferring 264 clinical staff and 39 beds to primary health level facilities. The transfer of excess clinical staff to those facilities which are closest to the communities may go a long way to reducing infant, child and maternal deaths in line with the Millennium Development Goals targets.

Nine (57.1 percent) of the 21 hospitals experienced MTFP deterioration during the three years. We found the sources of inefficiencies to be either adverse changes in pure efficiency, scale efficiency and/or technical efficiency.

In line with the "Health financing: A strategy for the African Region" [42], which was adopted by the 56^th ^WHO Regional Committee for Africa, Botswana needs to consider institutionalizing efficiency monitoring of health facilities within health management information systems (HMIS). Renner *et al *[45] proposes a number of steps that could be followed by countries that decide to institutionalize health facility efficiency monitoring within HMIS.

## Competing interests

The authors declare that they have no competing interests.

## Authors' contributions

Data was collected by NT and JN. NT, JN, LGS, EZA and JMK contributed to the design, analysis and writing of various sections of the manuscript. All authors read and approved the final manuscript.
